# Soon *PLOS Medicine* Will Be Ten: A Call for Papers on the Health of Pre-adolescent Children

**DOI:** 10.1371/journal.pmed.1001597

**Published:** 2014-01-28

**Authors:** The PLOS Medicine Editors

## Abstract

The *PLOS Medicine* editors issue a call for papers on the health needs of pre-adolescent children (5- to 10-year-olds) in the build up to *PLOS Medicine's* ten year anniversary later this year and explain why they want to highlight this neglected group of children.

*Please see later in the article for the Editors' Summary*

*PLOS Medicine* turns ten years old in October this year. As we approach this milestone, we could dwell on our substantial accomplishments over these first ten years, such as becoming the first high-impact open access peer-reviewed medical journal without pharmaceutical company advertising and without conflicts of interest in opinion-based articles [1]. However, instead, the *PLOS Medicine* editors wish to mark our tenth anniversary by drawing attention to the health and wellbeing of a neglected group—pre-adolescent children, including 10-year-olds, from around the world—by issuing a call for papers on research that addresses their health needs.

The UN typically defines adolescents as 10- to 19-year-olds [2]. But since the health needs of 10-year-old children are generally similar to those of pre-adolescents because most have not yet reached puberty, for the purposes of our ten year anniversary theme, we are considering pre-adolescent children as comprising the 5- to 10-year-old age group.

International attention on the Millennium Development Goals, particularly MDG 4, which aims to reduce by two-thirds 1990 mortality rates in children younger than 5 years by 2015, has led to much effort to implement effective interventions to reduce deaths in young children. Although more needs to be done, such efforts have had some success: in 2012 the global number of deaths in children aged under 5 years was the lowest in recent decades at 6.6 million [3], almost half the 1990 figure of 12.6 million [3].

With the international focus on child survival, many more young children will now live beyond their 5^th^ birthday and so enter into an age group that currently receives little attention. A recent UNICEF report calculates that over the past 22 years efforts to improve young child survival have saved around 90 million lives that might otherwise have been lost [3]. While this situation is encouraging, an important question remains: What do we know about the health of these 90 million children once they reach their 5^th^ birthday and beyond? Could they have survived 5 years only to still succumb to infectious diseases; injuries and trauma, such as drowning and road traffic crashes; non-communicable diseases, such as childhood cancers; or the effects of violence resulting from maltreatment or conflict? These conditions and situations are, unfortunately, common among many children but the international focus and attention on this pre-adolescent age group of children seems to be lacking. Do we have the data to show that deaths are also declining in this age group of children and that their lives are improving? In addition, are children from this age group working in adult roles and exposed to adult harms, rather than receiving an education? The advocacy for the needs and rights of this specific group of children is perhaps less than that for other groups.

At the other end of the age range bounded by puberty, the health of adolescents has recently received some attention. UNICEF's “The State of the World's Children 2011” report focused on adolescents and stated that, at that time, 1.2 billion individuals world-wide were aged 10–19 years [2]. And to coincide with last month's World AIDS Day, WHO and UNICEF issued a joint press release on the health needs of adolescents with HIV, highlighting that more than 2 million adolescents world-wide are living with HIV, many of whom do not receive the care and support that they need to stay in good health and prevent transmission [4]. WHO and UNICEF point out that adolescents are a neglected group with regards to HIV management, but pre-adolescent children are also receiving little attention in this regard. HIV-positive children in this younger age group also have to take HIV medicines for life, face stigma and discrimination, and are old enough to be involved in decisions regarding their own health.

Of course, we don't endorse a competition of which age group of children is the most neglected, or which should be highest priority—addressing the health needs of all children is of utmost importance. However, we are concerned about the lack of focus and data on the 5–10 year age group that currently falls between the under-fives and adolescents and are therefore using the opportunity of our tenth anniversary to encourage research into the health needs and health outcomes of this specific group of children.

We invite submission of research articles including clinical trials on new treatments for, and prevention of, common conditions affecting this age group; strong epidemiological assessments that provide more information about their health needs; and robust analyses of the impact of initiatives and interventions aimed at improving the health of pre-adolescent children. Authors should refer to the *PLOS Medicine* Guidelines for Authors at http://www.plosmedicine.org/static/guidelines.action for specific submission requirements, and all research papers should be submitted to *PLOS Medicine* with a clear statement in the cover letter that the submission is for consideration for our ten year anniversary theme.

We hope that you will join with us in highlighting the health needs of pre-adolescent children. We look forward to receiving your submissions.
